# Hæmato-Pyo-Metra in a Bitch

**Published:** 1882-10

**Authors:** 


### III.—HÆMATO-PYO-METRA IN A BITCH.

The cause of the distension in this instance is not very evident. Chronic endometritis does not often lead to it unless there is some narrowing at the os or in the vagina. From the amount of blood it may have been a hæmatometra at first, with subsequent inflammation of the mucosa, and suppuration.

On May 15th a spitz bitch, weighing seven and a half pounds, was brought to the Infirmary for treatment. No history could be obtained, save that for about ten days previous she had appeared dull, was thought to be constipated, and the belly had gradually enlarged.

On May 19th she was again brought to the Infirmary, and on being asked if he thought she could be in pup, the attendant said he was sure she could not be, but stated that some three weeks previous she was observed to be in heat, and that the flow, which was very great in amount, had suddenly stopped. After unsuccessfully endeavoring to dilate the os, an exploratory puncture was made through the walls of the uterus, with the result of obtaining a dark-brown fluid; a small canula was then inserted and about twelve ounces of this fluid withdrawn; a stimulant was given and the animal sent home. She died in about two hours after the operation was performed. The fluid contained some red blood corpuscles and many leucocytes-pus cells.

Post Mortem: Vagina looks normal. The os admitted a small knitting-needle. The body cavity seemed enlarged and contained a bloody fluid. A probe could be passed along the narrow neck into either cornu. The left was greatly dilated and contained a dark bloody fluid which, on standing, separated into a clear supernatant part, and a heavy grayish-red deposit, which was made up of pus and blood cells. The mucous membrane was rather thick, grayish in color, and presented numerous cubriform depressions and parallel ridges or elevations passing around at right angles to the axis of the cornu. The right cornu was slightly dilated; contained a similar kind of fluid. The mucous membrane presented numerous broad villous outgrowths. The ovaries were normal. The bladder and urethra normal.

MONTREAL, CANADA.

